# Glycogen Synthase Kinase-3

**DOI:** 10.4061/2011/279234

**Published:** 2011-12-11

**Authors:** Peter Crouch, Adam Cole, Michael Cousin, Ana Martinez, Katja Kanninen

**Affiliations:** ^1^Department of Pathology and Centre for Neuroscience, The University of Melbourne and Mental Health Research Institute, Parkville, VIC 3010, Australia; ^2^Neurosignalling Group, Garvan Institute for Medical Research, 384 Victoria St. Darlinghurst, Sydney, NSW 2010, Australia; ^3^Membrane Biology Group, Centre for Integrative Physiology, University of Edinburgh, George Square, Edinburgh EH8 9XD, UK; ^4^Instituto Quimica Medica (C.S.I.C.), Juan de la Cierva 3, 28006 Madrid, Spain; ^5^Department of Neurobiology, A.I.Virtanen Institute for Molecular Sciences, University of Eastern Finland, Kuopio, Finland

 Glycogen synthase kinase-3 (GSK3) is a ubiquitous and promiscuous kinase that has been studied extensively for over four decades. Initial reports beginning in the 1970s described its role in cellular metabolic pathways fundamental to glucose metabolism, but in more recent years the number of reports describing aberrant GSK3 activity in pathological conditions has risen dramatically.

 Interest in GSK3 in the field of Alzheimer's disease was first sparked in the early 1990s by papers that described the ability of GSK3 to phosphorylate tau. Excessive tau phosphorylation is present in Alzheimer's-disease-affected brain. These early papers provided new insight to the mechanisms that may contribute to tau pathology of Alzheimer's, with GSK3 as a potential central figure. Since then, the research effort invested into GSK3 in Alzheimer's disease has expanded, and mechanistic studies now demonstrate a functional relationship between not only GSK3 and tau, but also GSK3 and amyloid-*β*. Through weight of numbers, strong evidence now indicates that GSK3 is associated with the two key pathological features of Alzheimer's-disease-affected brain: neurofibrillary tangles and amyloid plaques.

 The scope of this special issue is to provide an overview of the data that implicate GSK3 in Alzheimer's disease. As an introduction, the special issue begins with a review of the regulation of GSK3 activity (M. Medina and F. Wandosell). This is followed by a report that provides caution by articulating the need to demonstrate the bona fide substrates of GSK3 (C. Sutherland). The role of GSK3 in the brain and neuronal function is then introduced by two reports. The first is on presynaptic function of GSK3 (K. J. Smillie and M. A. Cousin) and the second on GSK3 in brain development and neuronal plasticity (P. Salcedo-Tello et al.). After this the contribution of GSK3 to neurodegenerative diseases is described in four reviews. The first describes GSK3 in neurodegenerative diseases in general (P. Lei et al.) while the following three discuss more specific aspects of Alzheimer's disease, including cell survival mechanisms (M. A. Mines et al.), inflammation (j. Koistinaho et al.), and tau phosphorylation (D. P. Hanger and W. Nobel). Finally, the special issue concludes with two reviews on therapeutic strategies for Alzheimer's disease that focus on GSK3. The potential of targeting GSK3 for therapeutic benefit against oxidative stress is presented (K. Kanninen et al.), followed by an appraisal of GSK3 inhibitors in the next horizon (A. Martinez et al.).

 Since several GSK3 inhibitors are currently in clinical trials for treatment of neurological and other disorders, we feel this special issue is a timely “snapshot” of our current knowledge of GSK3 function in healthy and diseased brain, and highlights outstanding issues for future research on this important brain kinase.

*Peter Crouch* *Peter Crouch* *Adam Cole* *Adam Cole* *Michael Cousin* *Michael Cousin* *Ana Martinez* *Ana Martinez* *Katja Kanninen* *Katja Kanninen*

